# Alternative Anaphylactic Routes: The Potential Role of Macrophages

**DOI:** 10.3389/fimmu.2017.00515

**Published:** 2017-05-08

**Authors:** María M. Escribese, Domenico Rosace, Tomas Chivato, Tahia D. Fernández, Angel L. Corbí, Domingo Barber

**Affiliations:** ^1^Faculty of Medicine, IMMA Applied Molecular Medicine Institute, CEU San Pablo University, Madrid, Spain; ^2^Faculty of Medicine, Basic Medical Sciences Department, CEU San Pablo University, Madrid, Spain; ^3^Allergy Unit, Málaga Regional University Hospital-IBIMA, Málaga University, Málaga, Spain; ^4^Centro de Investigaciones Biológicas, CSIC, Madrid, Spain

**Keywords:** anaphylaxis, IgG, IgE, macrophages, serotonin

## Abstract

Anaphylaxis is an acute, life-threatening, multisystem syndrome resulting from the sudden release of mediators from effector cells. There are two potential pathways for anaphylaxis. The first one, IgE-dependent anaphylaxis, is induced by antigen (Ag) cross-linking of Ag-specific IgE bound to the high-affinity IgE receptor (FcεRI) on mast cells and basophils. The second one, IgG-dependent anaphylaxis is induced by Ag cross-linking of Ag-specific IgG bound to IgG receptors (FcγRI, FcγRIIA, FcγRIIB, FcγRIIC, and FcγRIIIA) on macrophages, neutrophils, and basophils. Macrophages exhibit a huge functional plasticity and are capable of exerting their scavenging, bactericidal, and regulatory functions under a wide variety of tissue conditions. Herein, we will review their potential role in the triggering and development of anaphylaxis. Thereby, macrophages, among other immune cells, play a role in both anaphylactic pathways (1) by responding to anaphylactic mediators secreted by mast cells after specific IgE cross-linking or (2) by acting as effector cells in the anaphylactic response mediated by IgG. In this review, we will go over the cellular and molecular mechanisms that take place in the above-mentioned anaphylactic pathways and will discuss the clinical implications in human allergic reactions.

## Pathway for Anaphylaxis: IgE and IgG Dependent

IgE-mediated anaphylaxis is well established and is thought to be the main anaphylactic pathway. However, increasing evidence obtained from animal models supports the existence of a second pathway. In this IgG-dependent pathway, macrophages instead of mast cells, and IgGs rather than IgE, are the immunoglobulins involved, and the main mediator released is platelet-activating factor (PAF) instead of histamine. Differences were detailed in Table 1. Data from IgG-mediated anaphylaxis were recopilated mainly from previous murine models, while data from IgE-mediated anaphylaxis were obtained from both animal and human previous reports (Table 1).

**Table 1:** **Main features in the mechanisms and triggering factors involved in IgE- and IgG-dependent anaphylactic pathways**.

	IgE-dependent pathway	IgG-dependent pathway
Ig involved	IgE	IgGs
Antigen concentration	Low	High
Fc receptor	FcεRI	FcγRI, FcγRIIA, FcγRIIB, FcγRIIC, FcγRIIIA, and FcγRIIIB
Effector cells	Mast cells	Macrophages, monocytes, and neutrophils
Mediators	Histamine (leukotrienes, prostaglandin, serotonin, etc.)	Platelet-activating factor (leukotrienes, prostaglandin, serotonin, etc.)
Triggering factors	Food, drugs (e.g., beta-lactam antibiotics), insect sting and bites, exercise (food dependent)	Food, drugs [monoclonal antibodies (omalizumab or infliximab)], or dextrans, others

In this review, we will analyze the evidence obtained from murine experimental models supporting the existence of an IgG-dependent anaphylaxis pathway and speculated about the possibility of a similar mechanism in humans, either as a stand-alone pathway or as a synergistic mechanism to IgE-mediated anaphylaxis.

The main body of evidence for IgG-mediated anaphylaxis comes from animal models.

Passive immunization, result of the administration of specific Igs, followed by enteral or parenteral challenge with the appropriate antigen (Ag) supported the relevance of IgE and mast cells in the development of anaphylaxis (1–3). Indeed, animals with depleted mast cells, IgE or FcεRI, subjected to active or passive immunization followed by oral challenge, completely suppress the anaphylactic reaction.

However, animal immunization followed parental challenge with the same Ag, revealed that anaphylaxis could even occur in the absence of the IgE/FcεRI/mast cell pathway. This demonstrates the existence of an alternative anaphylaxis pathway that closely resembles IgE-mediated anaphylaxis but involves other key players (3–5).

Both pathways display significant differences in their main features (Table 1), such as the requirement of different concentrations of Ag and Ab to induce the reaction.

In fact, studies comparing Ag doses required to elicit IgE- or IgG-mediated anaphylaxis suggested that the IgG-dependent pathway requires approximately 100-fold more Ag than the IgE pathway to induce a similar response (3).

Additionally, anaphylaxis mediated through IgG also appeared to require much more Ab than anaphylaxis mediated through IgE. In fact, IgE-mediated anaphylaxis can even be seen with serologically undetected sIgE levels, in which sIgE bound to mast cells is sufficient (5). In contrast, relatively high levels of serum IgG are required for Ag induction of anaphylaxis through the IgG pathway (3). This could be due to two factors: first, the much higher affinity of FcεRI for IgE than FcγRIII for IgG, and second, the fact that IgE binds directly to mast cell-associated IgE, whereas Ag/IgG complexes are presumably formed in blood and lymph before binding by FcγRIII on other immune cells such as macrophages (6, 7).

In the case of IgG-mediated anaphylaxis, the immunoglobulin subclasses and receptors involved in the reaction also play an important role. Regarding IgG subclasses, IgG1, IgG2a, and IgG2b have been reported to enable the induction of systemic anaphylaxis, inducing mild to severe hypothermia (8, 9). Furthermore, IgGs can bind to six different FcγR, namely, FcγRI, FcγRIIA, FcγRIIB, FcγRIIC, FcγRIIIA, and FcγRIIIB, which have different affinities, downstream signaling routes, and patterns of expression (10, 11). FcγRI is considered as the high-affinity receptor, although FcγRIIIB can bind IgG with high and low affinity depending on the IgG subclass (7).

Another crucial issue for development of either the IgE or IgG pathways of anaphylaxis is the balance between Ag concentration and the levels of IgG or IgE. Usually, both Ag-specific IgE and IgG are present in blood, with IgG levels being higher. Under these conditions, Ag will encounter IgG in blood before it can bind to mast cell-associated IgE, which results in blockage of IgE-mediated anaphylaxis. However, when Ag levels are insufficient to induce IgG-mediated anaphylaxis, high levels of IgG can prevent the development of any anaphylactic response. For a similar reason, larger amounts of Ag trigger anaphylaxis predominantly through the alternative pathway when Ag-specific IgG antibody levels are high, even though Ag-specific IgE is present. In this situation, the anaphylactic pathways will only be triggered simultaneously when the amount of challenge Ag exceeds the capacity of IgG antibody to block Ag binding to mast cell-associated IgE (5).

**Figure 1:**
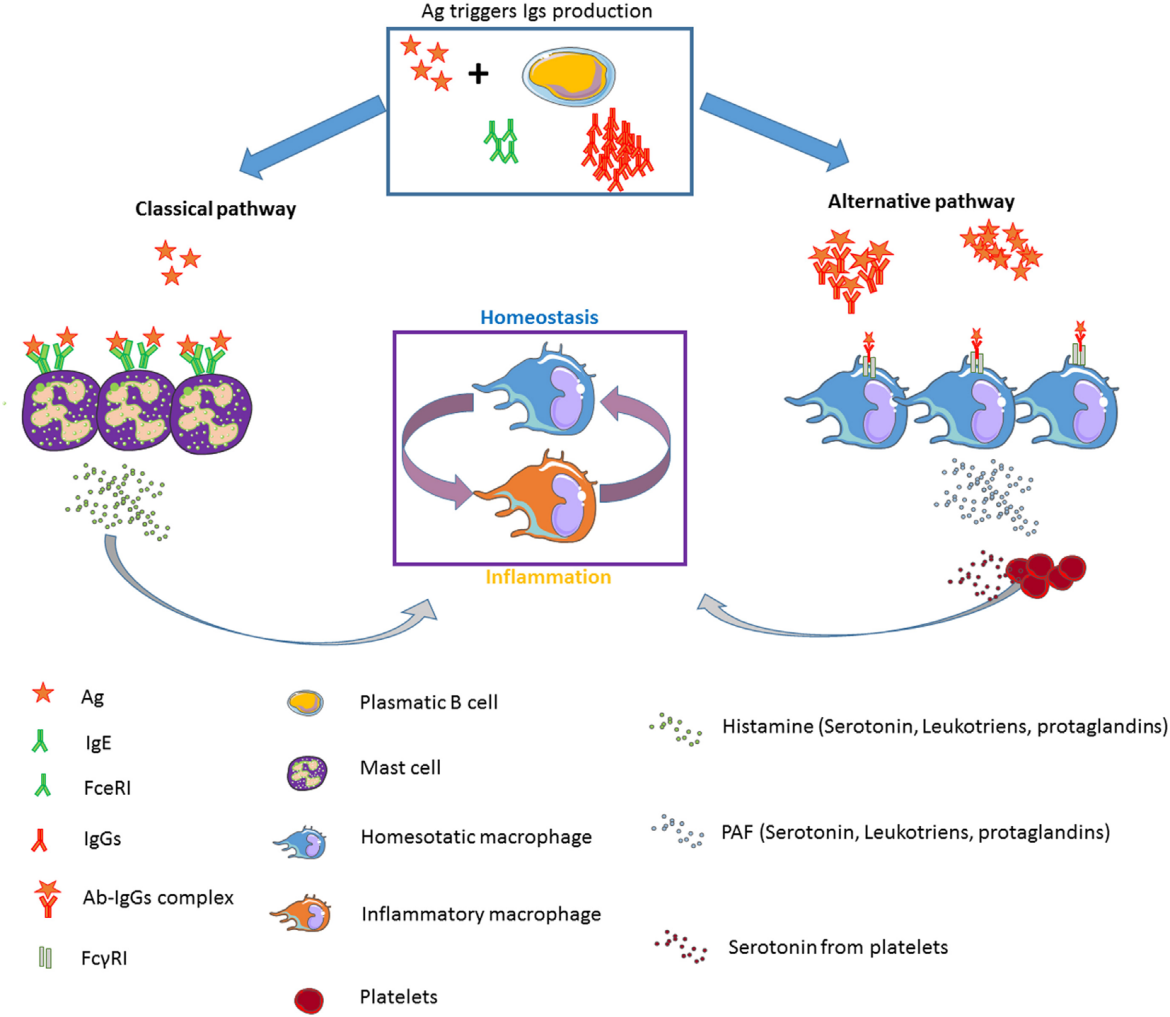
**Diagram showing the classical (IgE-dependent) and alternative (IgG-dependent) anaphylactic pathways: effector cells, mediators, Igs, and FcR implicated**.

Taken together, these data clearly support significant differences between both anaphylactic pathways regarding the type of Ig as well as the conditions needed for the development of one pathway or the other (Figure 1). However, in humans the relevance of the alternative pathway is still a matter of debate.

## Effector Cells and Mediators Involved in IgE- and IgG-Mediated Anaphylaxis

There is complete segregation of the effector cells and mediators underlying both anaphylactic pathways.

While it is well known that the IgE-dependent pathway of anaphylaxis is triggered by an allergen interacting with allergen-specific IgE bound to the FcεRI on mast cells, which leads to cross-linking and subsequent degranulation of the cells, the exact mechanism underlying the IgG anaphylactic pathway has not been completely elucidated. In fact, there is significant controversy about the effector cells involved in IgG-mediated anaphylaxis, and it seems that the main effector cells, at least in murine experimental models are macrophages/monocytes and basophils. However, some authors also suggest a role for neutrophils (8) and basophils (12). In fact, the latest publication of Khodoun et al., covering all three known effector cell types, concluded that all cells, monocytes/macrophages, basophils, and neutrophils, participate in IgG-induced anaphylaxis (13).

Another point of controversy is the level of FcγR expression and the type of myeloid cell expressing the receptor. In this regard, Beutier et al. showed that the differential expression of inhibitory FcγRIIB on myeloid cells and its differential binding of IgG subclasses control the contribution of basophils, neutrophils, and monocytes to IgG-dependent anaphylaxis, thus revealing novel complexities in cell population regulation mechanisms and, therefore, their relative contribution to IgG-induced reactions in murine models (14).

The outcome of this process of Ab–receptor recognition and subsequent cellular signaling activation is the release of several mediators responsible for the hypothermia and hypotension that characterize anaphylaxis.

The main mediator involved in IgE-mediated anaphylaxis is histamine. Histamine is known to play an essential role in the evolution of the anaphylactic process (15–17). Moreover, it is also involved in regulation of the immune response (18, 19). Other mediators released during IgE-mediated anaphylaxis are prostaglandins and leukotrienes (17, 20). Furthermore, a receptor for prostaglandins has also been described in several immune cells, such as macrophages or innate lymphoid cells (ILC2) (21).

Another significant metabolite reported to be released by mast cells upon IgE cross-linking is serotonin (22–24). The role of serotonin in the anaphylactic process is still unknown, although recent reports have suggested that this metabolite is key in immune response regulation (23, 25) and, more specifically, in the regulation of macrophage polarization and inflammatory resolution (26, 27), allergy (28), and hypotension (29). Serotonin participation in the regulation of inflammation and immune response upon anaphylaxis will be further discussed.

In the case of IgG-mediated anaphylaxis, the main mediator is PAF (5, 30, 31). It has been reported that serum PAF levels correlate with the severity of anaphylaxis (32). This metabolite is produced and secreted by several types of cells and is active at concentrations as low as 10^−12^ mol/L despite its short half-life (32).

Platelet-activating factor (PAF) is implicated in platelet aggregation and activation through the release of vasoactive amine during inflammatory responses, thus resulting in an increase in vascular permeability, circulatory collapse, a decreased cardiac output, and other biological effects (33).

Strikingly, platelets have been reported to be the major reservoirs of serotonin outside the nervous system (34), once again suggesting a novel role for serotonin in progression of the anaphylactic pathway as well as in allergic disease progression.

## Macrophages and Serotonin: Potential Novel Players in Anaphylaxis?

In anaphylaxis, macrophages have been described as effector cells in the IgG-dependent pathway, since they express FcγR and release PAF. This has been demonstrated in mouse models of anaphylaxis. Apart from this, no specific role has been described for these immune cells in IgE-dependent anaphylaxis in neither human nor mice. However, one could speculate that all the mediators released by mast cells (histamine, leukotrienes, and prostaglandin) might significantly affect macrophage polarization status and, thus, immune response outcome. These mechanisms will probably occur in both mouse and humans.

Macrophages and dendritic cells occupy a prominent position during immune responses, being essential for their initiation (a function primarily displayed by dendritic cells) and for the final effector phases (mostly macrophages) (35). In fact, and regardless of the triggering stimulus, macrophages are usually the final effectors of any given immune response, because they can acquire a continuum of functional states, thus adapting their effector functions to the surrounding environment and to the prevailing T cell-derived cytokines in the extracellular milieu receptor signals (36). By virtue of this plasticity, macrophages are not only critical for maintaining tissue homeostasis but can either display pro- or anti-inflammatory functions, promote or resolve an inflammatory response, and cause tissue damage or help in tissue repair. Results generated in recent years have clearly established the widespread homeostatic functions of macrophages, as they fine-tune physiological parameters as relevant as body temperature and even transit time in the gut (37–39).

Regarding factors with a prominent role in macrophage polarization and anaphylaxis, serotonin has also been shown to modify macrophage polarization in the phenotypic, cytokine, and transcriptional profile (27). Besides its production by mast cells (40), peripheral serotonin is mostly produced by enterochromaffin cells and later stored by platelets in dense granules (34). Serotonin not only promotes proliferation of numerous cell types but also functions as a regulator of immune and inflammatory responses. In fact, the immunomodulatory activity of serotonin is partly mediated through direct actions on macrophages: serotonin favors angiogenesis in colon cancer allografts by acting on tumor-infiltrating macrophages (41), contributes to pulmonary arterial hypertension by altering myeloid cell differentiation potential (42), and limits postoperative bowel inflammation *via* recognition by muscularis and peritoneal macrophages (43). At the molecular level, these actions appear to be mediated by serotonin receptors expressed on the macrophage cell surface. We have previously demonstrated that human anti-inflammatory macrophages specifically express HTR2B and HTR7 serotonin receptors, whose ligation results in altered macrophage transcriptome and inhibition of pro-inflammatory cytokine production (27). In fact, serotonin appears to switch the macrophage transcriptome toward a growth-promoting, anti-inflammatory, and pro-fibrotic gene profile, whose acquisition depends on both HTR2B and HTR7 (27). Therefore, we can speculate that agonists/antagonists of serotonin receptors might be therapeutically useful for limiting the uncontrolled production of pro-inflammatory cytokines that takes place in chronic inflammatory diseases (44). Surprisingly, HTR7 is the receptor responsible for serotonin-induced hypothermia (45), but whether macrophage HTR7 contributes to this response is currently unknown.

A reasonable hypothesis for the role of serotonin in the IgG-mediated anaphylaxis might be the generation of a feedback loop that favors the acquisition of an anti-inflammatory phenotype by macrophages right after the induction of an anaphylactic shock, aiming to restore homeostatic conditions (Figure 1).

Another strategy in line with the alternative anaphylactic pathway in humans that also supports the connection between changes in IgG concentration and a regulation of macrophages polarization is treatment with intravenous immunoglobulins (IVIg). IVIg is a preparation of polyclonal poly-specific IgG from the plasma of thousands of donors that is currently used as immunoregulatory and anti-inflammatory treatment in autoimmune and inflammatory disorders (46). The mechanism of action of IVIg has not been completely elucidated, but we have reported that IVIg skews human and mouse macrophage polarization through FcγR-dependent mechanisms (47). IVIg immunomodulatory activity is dependent on the macrophage polarization state, as it limits the pro-inflammatory nature of GM-CSF-dependent macrophages and favors the acquisition of pro-inflammatory properties in anti-inflammatory macrophages (47). In fact, IVIg enhances inflammatory tissue-damaging responses in murine models of stroke and sepsis and reduces tumor growth and metastasis by shifting the polarization state of tumor-associated myeloid cells toward the pro-inflammatory side (47). Since the latter effect was dependent on the expression of Fc receptors, we can conclude that ligation of molecules, such as CD16 and FcRγ, might be useful targets for the modulation of macrophage polarization.

## Evidence for IgG-Mediated Anaphylaxis in Human

The existence of IgG-mediated anaphylaxis in humans is not clear. In spite of a lack of direct evidence, the findings of some studies imply a possible alternative mechanism to IgE-mediated anaphylaxis (5, 30). PAF, which seems to be associated with the IgG mechanism in mice, is an essential mediator in human anaphylaxis, and its levels are elevated in patients undergoing anaphylaxis compared with a control group (48). The catabolism of this mediator is controlled by the enzyme PAF acetylhydrolase (PAF-AH), which is in charge of PAF inactivation (49). Some studies have correlated the levels of these two markers with the severity of anaphylaxis, with increases in PAF levels and decreases in PAF-AH activity. Moreover, patients with the lowest levels of PAF-AH activity were found to exhibit a 27-times higher risk of developing severe or fatal anaphylaxis than patients with normal levels (48, 50).

Several cases of drug anaphylaxis are classified as non-allergic due to the absence of specific IgE titers (measured in sera or by skin test) and the lack of increased serum tryptase or basophil activation (51), although no study has addressed the IgG-mediated mechanism in these patients.

However, in patients treated with biological drugs, these can induce anaphylaxis without the presence of detectable specific IgE, although they do present high levels of specific IgG (52). This observation derives from patients with IgA deficiency who developed anaphylaxis after receiving a blood transfusion or treatment with intravenous injections of IgA. In these subjects, increased levels of IgG anti-IgA antibodies were also found (53, 54).

Moreover, in a later study patients with higher levels of IgG were found to present an increased frequency of a gain-of-function allele of the stimulatory FcγRIIA (55), although this study was conducted in a limited number of subjects.

The presence of increased titers of specific IgG has also been observed in patients treated with human, humanized, or chimeric mAbs, such as infliximab or adalimumab (56), and other biological factors (57–59). In the case of infliximab, the presence of high levels of specific IgG has been related to an increased risk of suffering anaphylaxis (60). A common factor to all these reported cases was the administration of high quantities of the suspected Ag, leading to the presence of high levels of specific IgG.

As with drug allergies, evidence for the existence of IgG-mediated anaphylaxis has also been found in cases of food allergy, especially in anaphylaxis induced by lipid transfer proteins (LTP). Increased levels of anti-LTP IgG1 and IgG3 and increased expression of the three genes coding for the activating receptor FcγRI (CD64) have been observed in a group of patients with food anaphylaxis induced by LTP (61). Mast cells can be activated by IgG *via* this receptor (62, 63) and are able to recognize both IgG1 and IgG3 with high affinity (64, 65). Interestingly, both anti-LTP specific IgG and IgE have been found in LTP allergic patients, which could suggest an involvement of both pathways in the anaphylactic mechanism in these subjects (61). The most severe food allergens are milk, egg, and peanut, and all of them share a high allergenic concentration, thus fulfilling the criteria necessary to elicit an alternative anaphylactic pathway.

## Conclusion and Further Expectations

Anaphylaxis is the most serious allergic reaction that can occur and may even endanger the patient’s life. Moreover, epidemiological data indicate that cases of anaphylaxis are increasing worldwide. The mechanisms involved in the pathogenesis of anaphylaxis can be immunological or non-immunological. Classical immunological reactions mediated by IgE are observed in food anaphylaxis, beta-lactam antibiotics, or hymenopteran stings. Immunological reactions mediated by IgG are being observed following administration of certain monoclonal antibodies (omalizumab or infliximab) or dextrans. The role of macrophages is relevant in this type of IgG-mediated immunological anaphylaxis. PAF released by activated macrophages can activate mast cells, explaining the pathogenesis of this anaphylaxis. Given the increased use of different monoclonal antibodies in clinical practice for the treatment of immune-based diseases, an increase in this type of IgG-mediated anaphylaxis might be observed.

## Author Contributions

All the authors have participated in the writing of the manuscript. DB has written, organized, and revised the manuscript.

## Conflict of Interest Statement

The authors declare that the research was conducted in the absence of any commercial or financial relationships that could be construed as a potential conflict of interest.
